# Erratum to “Cutaneous manifestations of coronavirus disease 2019” by Ghafoor, Ali, and Goldust (2022)^1^

**DOI:** 10.1111/jocd.15656

**Published:** 2023-01-30

In Ghafoor, Ali, and Goldust.,^1^ the degree for the second author has been corrected as follows:

Syeda Mahanum Ali, MCPS
